# What you listen to makes a difference: The impact of music on attention and well‐being

**DOI:** 10.1111/nyas.70049

**Published:** 2025-09-10

**Authors:** Lucia De Francesco, Selene Schintu, Alessandro Mazza, Raffaella Ricci, Olga Dal Monte

**Affiliations:** ^1^ Department of Psychology University of Turin Torino Italy; ^2^ Center for Mind/Brain Sciences—CIMeC University of Trento Rovereto Italy; ^3^ Department of Psychological and Brain Sciences The George Washington University Washington District of Columbia USA; ^4^ Neuroscience Institute of Turin—NIT Torino Italy

**Keywords:** attentional network, brown noise, executive network, fast beat music, slow beat music, sound

## Abstract

Sounds constantly surround us, serving as sensory cues that help humans interpret the world and navigate the flood of stimuli they encounter. Research has shown that sounds and music can influence attentional performance; however, evidence on whether auditory stimuli can improve attention is limited. This study employed the attention network test to investigate how four types of sound—slow beat music, brown noise, fast beat music, and no sound—modulate visual attention. Moreover, we assessed the impact of auditory stimuli on physiological response (i.e., heart rate) and subjective experience. Results indicate that slow beat music has several positive effects: it enhances the efficiency of attentional orienting and the ability to focus on relevant targets while ignoring distractors, lowers heart rate, and increases subjective feelings of pleasure and relaxation. The benefits of slow beat music on attentional performance and psycho‐physiological state suggest that it can be used to enhance focus, as well as reduce the risk of errors and stress in attention‐demanding contexts.

## Graphical abstract

This study examines how slow beat music, fast beat music, and brown noise modulate attention, heart rate, and subjective experiences. Slow beat music improved attentional orienting, helped focus on targets while ignoring distractors, lowered heart rate, and increased the subjective feeling of relaxation and pleasure. These results suggest that slow beat music can enhance focus and reduce stress and errors in tasks that demand high attention.

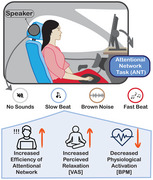

## INTRODUCTION

Imagine driving home, listening to your favorite fast beat song at a fairly high volume to decompress after a long day at work. At some point, unexpected roadwork forces you to take a detour, and you find yourself on an unfamiliar road. You will likely find yourself turning down the volume to focus and pay more attention to the road and navigate your way home. Indeed, selecting salient external information for guiding our actions and optimally navigating our environment is what defines attention.^1^ As we can infer from our initial example, not only is attentional capacity limited, but also affected by concurrent salient stimuli, even if presented in a different modality. In such demanding contexts, attention integrates inputs from multiple sensory modalities. Thus, visual attention may be influenced by different types of stimuli that are present in our surroundings. For example, it has been shown that concurrent presentation of salient auditory stimuli impairs visual attention, and vice versa.^2^ Additionally, background noises, like urban environments, increase mental workload and decrease visual/auditory attentional capacity. Moreover, auditory‐vocal noises increase drivers’ reaction times.^3^ However, are sounds always detrimental to attentional capacity? Or could certain sounds potentially enhance cognitive functions?

Many daily life activities, such as driving or cycling, demand attention to numerous bottom‐up stimuli across various sensory modalities, along with top‐down processes such as motor execution and spatial orientation.^4^ The attentional orienting network is responsible for selecting relevant stimuli in the surrounding environment (i.e., pedestrians, signals, other cars) and re‐orienting attention as needed in response to salient auditory or visual stimuli. Meanwhile, the executive network enables the selection and extraction of relevant information while filtering out distractors that could impair performance. The efficient interplay of these high‐level cognitive processes is crucial to guarantee the correct completion of a task and reduce the error rate, which previous driving example, translates into increased safety.^5^ Therefore, the possibility for auditory stimuli to increase the efficiency of the attentional network is of particular interest. However, there is currently little and contradictory data regarding the possible benefits of certain types of sound on human attention.

Concerning low‐level auditory stimuli at specific frequencies, white noise has been found to positively affect attentional and memory performance,^6^, ^7^ whereas the effects of brown noise on cognition have been scarcely investigated.^8^ On the other hand, the impact of high‐level auditory stimuli, such as music, on attention has been extensively studied. This line of research is grounded in the arousal‐mood hypothesis, which suggests that background music influences cognitive performance by modulating arousal and mood, which in turn affect attention.^9^ This model posits that specific brain networks mediate the influence of background music on selective attention,^10^ and emotional states induced by music, such as happiness or sadness, in turn, affect alerting, orienting, and executive attention.^11^ However, findings supporting such a model are inconsistent, with some suggesting no mood‐induced changes in attention and others highlighting how emotional content, such as happy or sad music, can modulate different types of attention.^12^, ^13^ The picture is further complicated by evidence that music enhances attention solely when characterized by certain features, such as a major key, upbeat tempo, and cheerful melodies,^14^, ^15^ and that individual differences, such as personality traits or tempo preference, interact with music to influence cognitive performance.^16^ ^,^ ^17^ Therefore, although some studies have reported improved attentional performance while listening to slow beat music,^12^ this area of research still lacks a systematic examination of the effects of music that is disentangled from emotional content. Investigating whether a direct physiological link exists between arousal and attention, independently of the subjective measure of mood, would be crucial for advancing our understanding of how music influences attentional processes.

It is well‐established that various contexts trigger specific physiological activations and associated stress responses,^18^, ^19^ and music is no exception. Relaxing music, typically with 66–76 beats per minute (BPM), reduces stress, anxiety, cortisol levels, heart rate, and blood pressure.^20^, ^21^, ^22^, ^23^ In contrast, faster beat music, with ∼200 BPM, has been shown to boost energy level, especially during physical activities.^24^, ^25^ Although understanding the impact of music on psycho‐physiological states and attention has generated interest, the results of the impact of sound on attention remain inconclusive.^26^ This may be due to the lack of a direct comparison of different types of music or noise, as well as inconsistencies in research focus; some studies examine background music's effects on attention, whereas others assess music as a main activity in relation to arousal without integrating these perspectives. Additionally, results often vary due to the use of different measures to assess arousal, sometimes relying solely on self‐reports rather than physiological indicators.

To address this gap, the present study investigates the effect of background noise and music on attentional performance, as well as the impact of these auditory stimuli on psycho‐physiological responses. By directly comparing multiple auditory conditions and integrating subjective and physiological measures, this study offers a multifaceted perspective on how different sounds influence cognitive and physiological processes. We adopted a car‐like setting to represent an environment that demands a high level of attention and significant cognitive effort while also potentially inducing stress. We employed the attention network test (ANT)^27^ to quantify participants’ attentional performance when exposed to different types of auditory stimuli (*no sound*, *brown noise*, *slow beat*, and *fast beat* music). Additionally, we quantified the impact of the different auditory stimuli at both implicit (i.e., physiological reaction measured by heart rate) and explicit (i.e., subjective experience measured by subjective ratings) levels. Based on previous findings, we expect the slow‐beat music to have a relaxing effect, as reflected in both physiological and subjective responses. Additionally, we anticipate that this effect will be accompanied by an increase in attentional performance efficiency.

## MATERIALS AND METHODS

### Participants

Twenty‐six adults with normal or corrected‐to‐normal vision and no history of neurological or hearing problems participated in the study (11 females; age 22.57 ± 3.14 SD). A priori power analyses using GPower*3 indicated that to achieve an 85% statistical power for detecting a medium effect size (*f* = 0.25) with an *α* error level of 0.05, a sample size of *N* = 26 was required. Participants were recruited from a university database or through advertisements posted on the University of Turin website. Participants signed informed consent and did not receive any compensation. The study was approved by the Bioethical Committee of the University of Turin and conducted following the ethical standards of the 2013 Declaration of Helsinki (World Medical Association; 2013).

### Procedure

**FIGURE 1:**
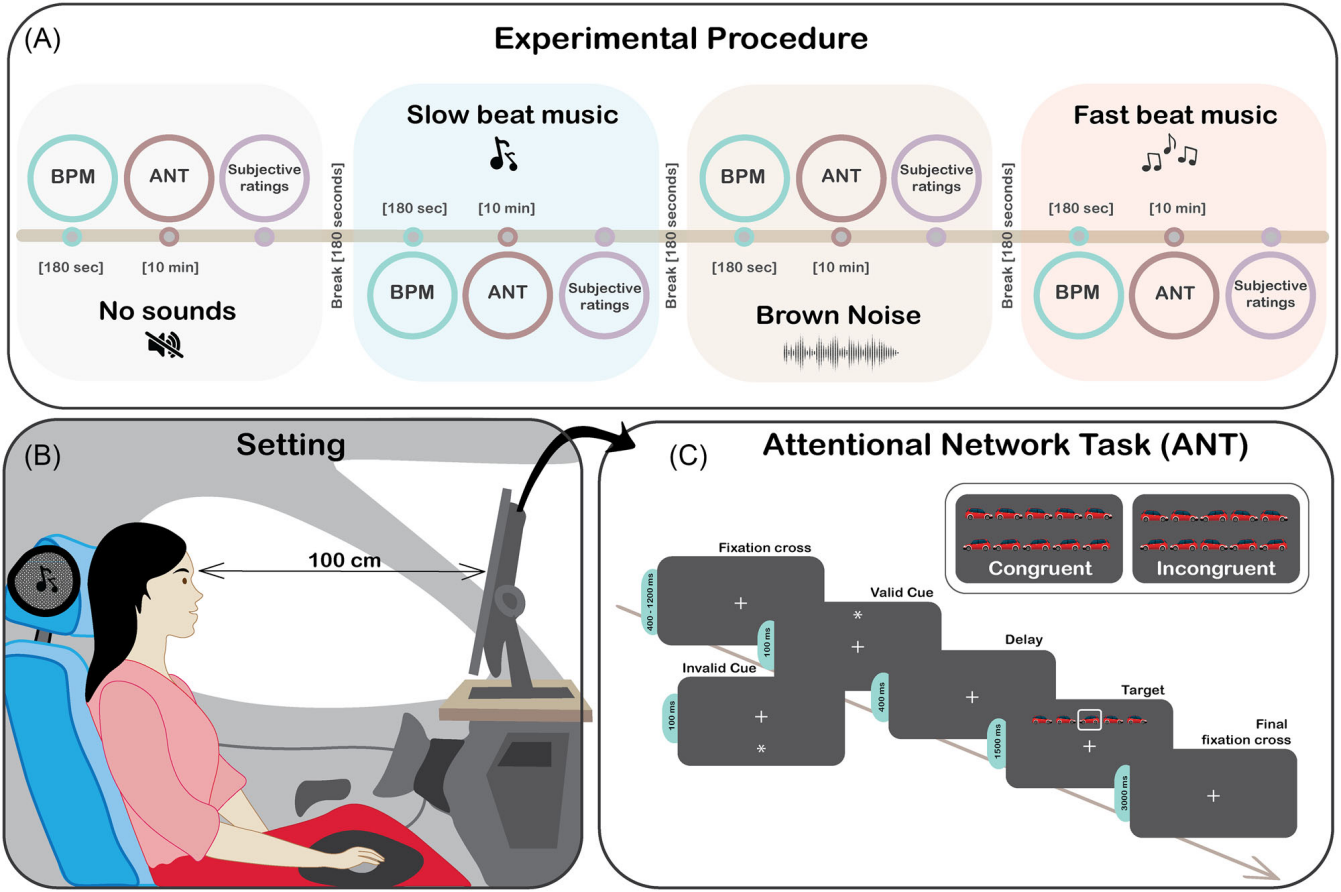
Experimental setting, task, and experimental procedure. (A) Experimental procedure. Each condition started with the recording of the participant's physiological activity at rest while hearing the sound (180 s). Participants then completed the attention network task (ANT; 10 min) to quantify attentional performance and rated their subjective impact of the auditory stimulation. The sound was present during the whole condition, a 2‐min break separated the four conditions (*no sound*, *slow beat*, *brown noise*, and *fast beat*), and the order of the conditions was counterbalanced across participants. (B) Setting of the experiment. For the whole duration of the experimental procedure, participants were comfortably seated inside a car‐like environment, which was comparable to a real car interior. Participants were exposed to the auditory stimuli through a Bluetooth device placed on the headrest. The ANT was presented on a black screen (27″) placed 100 cm from the participant's eyes, and the mouse used to collect responses was placed on a handrest located on the participant's right hand side. The position of the mouse aligned with the gear shift location of this car model. (C) ANT procedure. Each trial started with a fixation cross in the middle of the screen, which was presented for 400–1200 ms. A spatial cue (asterisk) then appeared for 100 ms above or below the fixation cross. After 400 ms a line of five cars appeared in the same position of the cue (*valid trials*) or in the opposite one (*invalid trials*). The other four cars of the line could be facing the same direction as the central car *(congruent trials*) or the opposite one (*incongruent trials*).

The experiment consisted of four consecutive conditions, carried out with four different auditory stimulations (*no sound*, *slow beat*, *brown noise*, *fast beat*; Figure 1). Each condition started with the recording of participants’ physiological activity at rest while hearing the auditory stimulus (180 s). They then completed the ANT (10 min) to assess their attentional performance and rated the impact of the auditory stimulation (subjective ratings). The sound was present throughout the whole condition, with a break of 180 s between conditions. The order of the conditions was counterbalanced across participants (Figure 1A).

Throughout the whole experiment, participants were comfortably seated in a car‐like environment, which was comparable to a real car interior (Figure 1B). The environment was enclosed by two black bulkheads to isolate the participants from possible background noise and control for light exposure. Participants listened to the auditory stimuli through a Bluetooth speaker placed on the headrest. The ANT (Figure 1C) was presented on a black screen (27″) placed 100 cm from the participants’ eyes, and the mouse used to collect responses was on a handrest placed on the participants’ right side.

### Auditory stimuli

Besides the condition completed in silence (*no sound*), participants completed the task under three auditory conditions: one noise (*brown noise*) and two melodies (*slow beat*, about 50 BPM; *fast beat*, about 155 BPM) to compare the effects of music and noise, which are often studied separately in the literature. Brown noise was selected as a low‐level auditory control to differentiate the effects of music from non‐musical auditory stimulation. It was preferred over white noise because both have been shown to produce similar effects, but brown noise has been less investigated in behavioral research, thus contributing to expanding the literature in this area. We then selected two types of music with distinct tempos: a slow beat song (around 50 BPM), known to promote relaxation, and a fast beat song (around 155 BPM), which is typically associated with increased energy. Although previous studies have highlighted the different effects of these types of stimuli on relaxation and energy, their impact on attention and performance remains unclear.^24^ The audio stimuli were not linked to a particular genre of music or mood to prevent the influence of other confounding factors. The BPM of a song was calculated based on how many times the primary beat occurs within 1 min. Sounds were delivered through the speaker placed on the head rest, with the volume set to a consistent level across all conditions to ensure that the sounds were presented at the same intensity (Figure 1B). The device was controlled with MATLAB, which determined when to turn on or off the sounds during the experiment.

## ASSESSMENT

### Physiological recordings

An MP150 (Biopac Systems Inc.) biosignal amplifier was used for electrocardiography (ECG) to quantify changes in heart rate. Pre‐gelled shielded electrodes were applied with a Lead II montage using the standard limb electrode placement.^28^ The gain parameter was set at 1000 (±10 mV), and the signal was sampled at 500 Hz with a 150 Hz low‐pass and a 0.05 Hz high‐pass filter. ECG data were processed using custom scripts on MATLAB (version 2021a, The MathWorks, Inc.). Although ECG data were collected continuously throughout each condition, we extracted BPM specifically from the 180‐s resting period that preceded the ANT task. This period was selected because it involved passive listening with minimal movement, which reduced the likelihood of artifacts affecting BPM measurement.

### ANT

We employed a 10‐min version of the ANT to quantify the orienting and the executive network.^27^ This version was chosen to keep the entire experimental procedure duration within 1 h and thus prevent fatigue that could affect measurement quality. Each trial started with a fixation cross appearing in the middle of the screen for 400–1200 ms. A spatial cue (asterisk) then appeared for 100 ms above or below the fixation cross. After 400 ms, a line of five car icons appeared in the same position as the cue (*valid trials*) or in the opposite one (*invalid trials*). Participants were instructed to respond according to the direction of the central car by pressing the right mouse button if it was facing right and the left button if it was facing left. They were asked to complete the task as accurately and quickly as possible. The other four car icons could be facing the same direction as the central car (*congruent trials*) or the opposite one (*incongruent trials*) (Figure 1C).

The first portion of the task quantifies the orienting network efficiency—the ability to orient attention—measured by the response times (RTs) of *valid trials*, and the ability to re‐orient attention, measured by the RTs of *invalid trials*. The second portion of the task quantifies the executive network efficiency, the ability to respond to the target influenced by facilitators (RTs in *congruent trials*) and distractors (RTs in *incongruent trials)*. Before the start of the first experimental condition, participants underwent a short practice session of the ANT (six trials).

### Subjective ratings

At the end of each condition *(no sound, slow beat, brown noise, fast beat*) and after completing the ANT, we asked participants to quantify the perceived impact of the auditory stimuli on a visual analogue scale ranging from −10 to 10. Participants were asked to quantify (1) pleasantness (*How pleasant did you perceive this sound?)*, (2) sense of relaxation *(How much did you feel relaxed by this sound?)*, (3) level of concentration (*How much did you feel concentrated by this sound?)*, (4) level of wakefulness *(How much did you feel awakened by this sound?)*, and (5) familiarity (*How familiar was this sound?)* of the stimuli.

### Statistical analyses

Statistical analyses were performed using MATLAB (version R2024b) and SPSS (IBM, version 27.0) with an alpha set at 0.05. In order to take into account individual differences, the data (subjective ratings, RTs, and BPM) were first *z*‐scored using the mean and standard deviation of the four conditions of each subject. Concerning the ANT, we considered RTs only for correct responses. Two‐tailed paired FDR‐corrected *t*‐tests were carried out as post hoc comparisons. Effect sizes are indicated for significant effects.

## RESULTS

### Attention: Orienting and executive network

**FIGURE 2:**
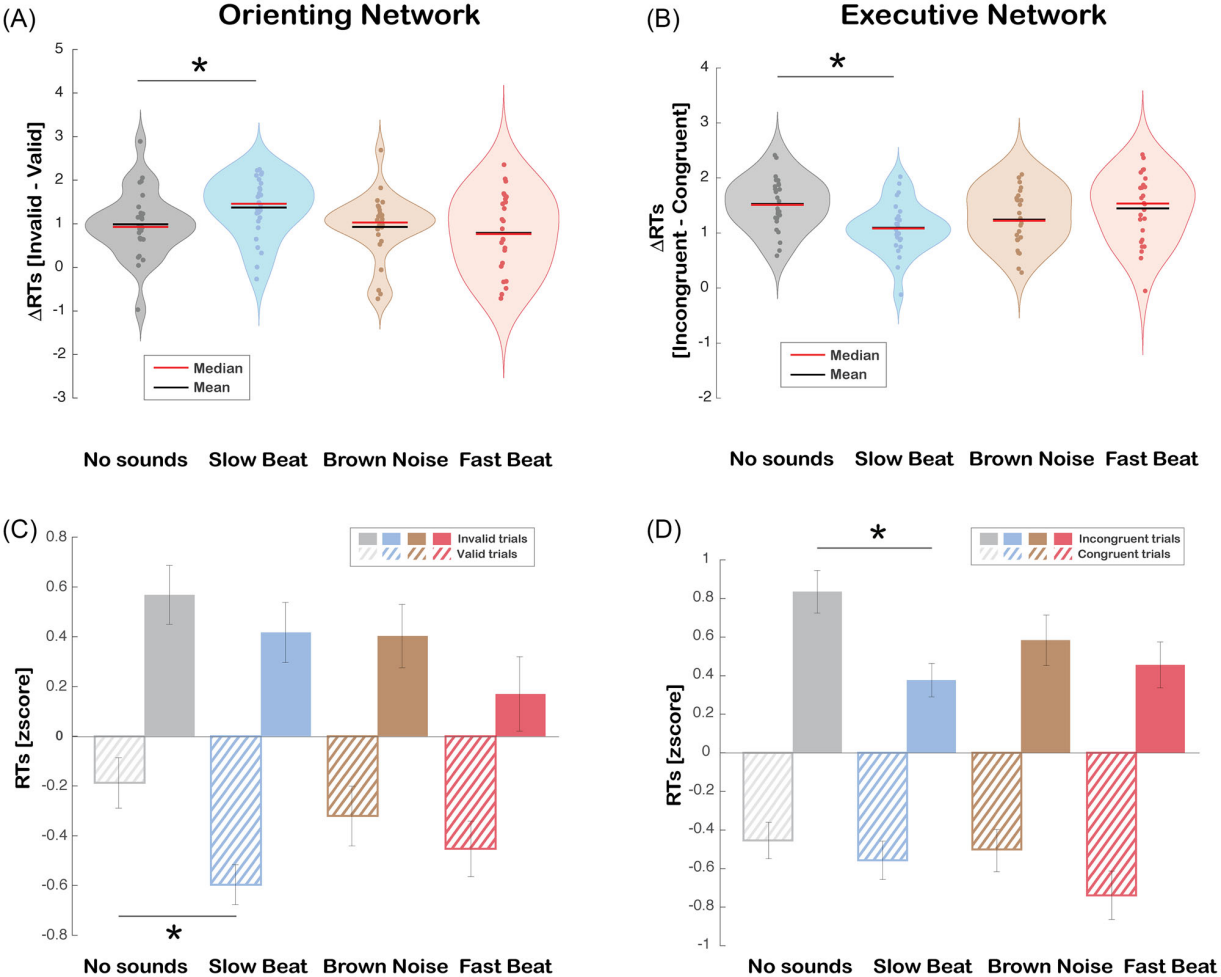
Effects of sounds on the orienting network and executive network. (A) Violin plots representing the distribution of *Δ* mean [RTs invalid—RTs valid] across four conditions (*no sound*, *slow beat*, *brown noise*, *fast beat*). The width of each violin corresponds to the density of data points, with thicker areas indicating higher concentrations of values. The red line represents the median, the black line indicates the mean, and dots represent individual data points, showing the spread of values. (B) Violin plots representing the distribution of *Δ* mean [RTs incongruent—RTs congruent] across four conditions (*no sound*, *slow beat*, *brown noise*, *fast beat*). The width of each violin corresponds to the density of data points, with thicker areas indicating higher concentrations of values. The red line represents the median, the black line indicates the mean, and dots represent individual data points, showing the spread of values. (C) Mean *z*‐score RTs for the orienting network across four experimental conditions (*no sound*, *slow beat*, *brown noise*, *fast beat*), with *valid trials* generally showing faster responses relative to *invalid trials*, and *slow beat* music reducing the RTs compared to *no sound* in *valid trials*. Error bars indicate the standard error of the mean (SEM). (D) Mean *z*‐score RTs for the executive network across four experimental conditions (*no sound*, *slow beat*, *brown noise*, *fast beat*), with *congruent trials* generally showing faster responses relative to *incongruent trials*, and *slow beat* music reducing the RTs compared to *no sound* in *incongruent trials*. Error bars indicate the standard error of the mean (SEM). *p < 0.05.

To determine the influence of four types of sound on the two attentional networks, we first computed the *Δ* of the RTs for the orienting and the executive network. The one‐way repeated measures ANOVA on the orienting network  (*Δ* RTs = RTs invalid trials ‐ RTs valid trials) with sound (no sound, slow beat music, brown noise, fast beat music) as the within‐subjects factor was found to be significant [*F*_(1, 25)_ = 4.925, *p* = 0.003, $\eta_{p}^{2}$ = 0.165]. Post hoc comparisons showed that *Δ* RTs for slow beat music (*M* = 1.429, SEM = 0.145) was significantly greater than the no sound condition (*M* = 0.965, SEM = 0.150, *t*_(25)_ = −2.603 *p* = 0.04, all other *p*s > 0.05), indicating that there was a bigger difference between valid and invalid (Figure 2A). The same approach was used for the executive network, where the *Δ* was calculated as the difference in RTs between incongruent and congruent trials (*Δ* RTs = RTs incongruent trials ‐ RTs congruent trials) to measure the interference effect. A one‐way repeated measures ANOVA on the interference effect also revealed a significant main effect of sound [*F*_(1, 25)_ = 4.317, *p* = 0.007, $\eta_{p}^{2}$ = 0.147]. Post hoc comparisons showed that slow beat music (*M* = 1.091, SEM = 0.091) was significantly smaller than the no sound condition (*M* = 1.527, SEM = 0.092, *t*_(25)_ = 3.467, *p* = 0.01, all other *p*s > 0.05), indicating a decreased *Δ* for the music condition, and thus a smaller difference between congruent and incongruent (Figure 2B).

Given that we found interesting differences in *Δ* between the no sound and the slow beat condition, we were interested in understanding which trials drove these results. Specifically, we wondered whether the increased difference between invalid and valid trials was determined by either an increase in RTs during invalid trials or a decrease RTs during valid trials. Moreover, the reduction in the *Δ* between incongruent and congruent trials could have been determined by an increase in RTs during congruent trials or a decrease in RTs during incongruent trials. To have a complete picture and answer to those questions, we used *z*‐scored RTs in a repeated measures ANOVA having orienting network (*valid trials* and *invalid trials)*, executive network (*congruent trials* and *incongruent trials)*, and sound (*no sound*, *slow beat*, *brown noise*, *and fast beat*) as within‐subject factors. As expected from previous studies, we found main effects for the orienting network [*F*_(1, 25)_ = 76.394, *p* < 0.001, $\eta_{p}^{2}$ = 0.753] and the executive network [*F*_(1, 25)_ = 383.787, *p* < 0.001, $\eta_{p}^{2}$ = 0.939], along with the interaction between these two factors [*F*_(1, 25)_ = 14.640, *p* < 0.001, $\eta_{p}^{2}$ = 0.369]. Indeed, subjects were faster to answer *valid trials* (*M* = −0.392, SEM = 0.045) compared to *invalid trials* (*M* = 0.392, SEM = 0.045, *t*_(25)_ = −8.740, *p* < 0.001). Faster responses were found in *congruent trials* (*M* = −0.553, SEM = 0.028) compared to *incongruent* (*M* = 0.553, SEM = 0.028, *t*_(25)_ = −19.590, *p* < 0.001), and this difference between *congruent* and *incongruent* was higher in *invalid trials* [RTs *invalid congruent* (*M* = −0.243, SEM = 0.059) RTs *invalid incongruent* (*M* = 1.027, SEM = 0.056, *t*_(25)_ = −17.748, *p* < 0.001)], which determined the interaction effect between the orienting and executive network. These effects indicate that the task was well implemented, as subjects were influenced by the cue position or by the presence of distractors. The same analyses showed that the main effect of sound did not reach significance (*p* > 0.05); however, the interaction effects between sound and the orienting network [*F*_(1, 25)_ = 2.888, *p* = 0.04, $\eta_{p}^{2}$ = 0.104] and between sound and the executive network [*F*_(1, 25)_ = 4.899, *p* = 0.003, $\eta_{p}^{2}$ = 0.164] were significant. The follow‐up analysis on sound and orienting network interaction revealed a greater reduction in RTs for *slow beat music* (*M* = −0.205, SEM = 0.103) compared to the *no sound* condition (*M* = −0.589, SEM = 0.082) when subjects responded to *valid trials* (*t*_(25)_ = −2.915, *p* = 0.04; all other *p*s > 0.05; Figure 2C). Such RTs reduction in *valid trials* during the exposure to the *slow beat music* indicates a faster speed processing and greater automatic attention, thereby an enhanced ability to accurately respond to a target when preceded by a signal. Post hoc comparisons following the interaction between sound and executive network revealed a reduction in RTs during *slow beat music* (*M* = 0.368, SEM = 0.086) compared to the *no sound* condition (*M* = 0.824, SEM = 0.109) when subjects responded to the *incongruent trials* (*t*_(25)_ = −3.126, *p* = 0.027; all other *p*s > 0.05; Figure 2D). Such reduction in RTs during exposure to the slow beat music in *incongruent trials* indicates a minor influence of the distractor in completing the task and thereby a greater executive network efficiency.

### Physiological activation

**FIGURE 3:**
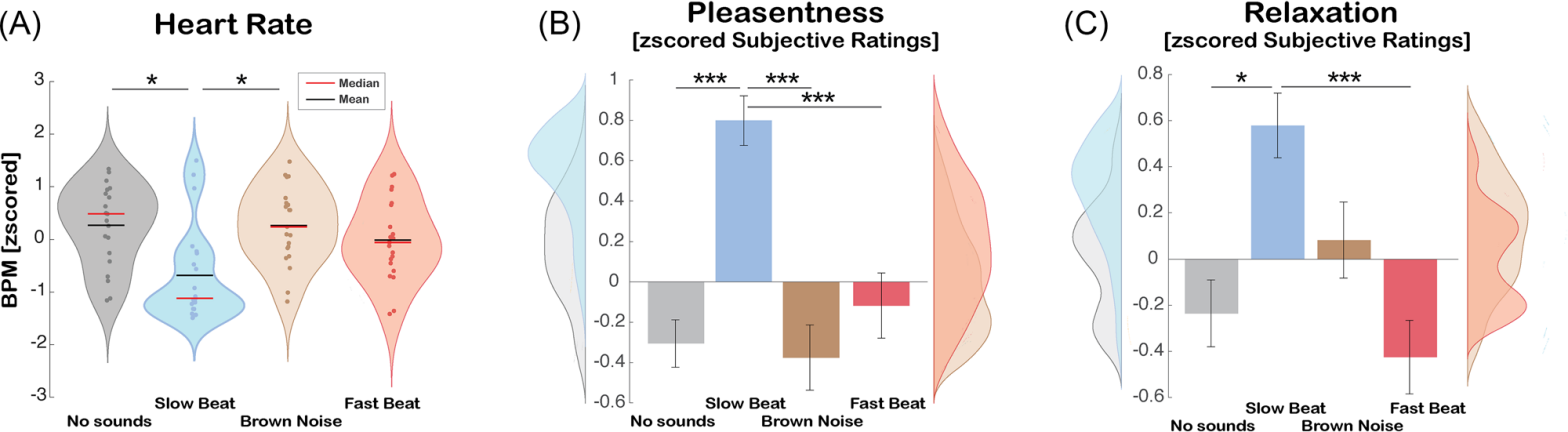
Effects of sounds on heart rate and subjective ratings. (A) Violin plots representing the distribution of beats per minute (BPM) across four conditions (*no sound*, *slow beat*, *brown noise*, *fast beat*), with *slow beat* music showing a reduced response compared to other conditions and significantly lower compared to *no sound* and *brown noise*. The width of each violin represents the density of data points within each condition. Dots represent individual data points, showing the spread of values. (B) Bar plot with associated distributions representing pleasure levels across four experimental conditions (*no sound, slow beat, brown noise, fast beat*). The bars show the *z*‐score means with standard error, whereas the side distributions illustrate data variability for each condition. Participants rated the *slow beat* music as more pleasant compared to all the other conditions. (C) Bar plot with associated distributions representing relaxation levels across four experimental conditions (*no sound, slow beat, brown noise, fast best*). The bars show the *z*‐score means with standard errors, while the side distributions illustrate data variability for each condition. Participants rated the *slow beat* music as more relaxing compared to *no sound* and *fast beat* music. *p < 0,05; ***p < 0,001.

To quantify the impact of the four different types of sound on participants' arousal, we extracted and analyzed heart rate (BPM) during the first 180 s of each condition. Due to technical issues, five participants’ data were corrupted, yielding to a final sample of 21 participants. The BPM was analyzed using a repeated measures ANOVA with sound (*no sound*, *slow beat*, *brown noise*, *fast beat*) as within‐subjects factors. The post hoc comparisons following the significant main effect of sound [*F*_(1, 20)_ = 4.726, *p* = 0.005, $\eta_{p}^{2}$ = 0.191] showed that *slow beat* (*M* = −0.678, SEM = 0.198) decreased BPM compared to *no sound* (*M* = 0.270, SEM = 0.166; *t*_(20)_ = −2.945, *p* = 0.024) and *brown noise* (*M* = 0.267, SEM = 0.158; *t*_(20)_ = −3.019, *p* = 0.040; Figure 3A). The exposure to the *slow beat* was the only condition decreasing the physiological activation compared to the absence of music (all other *p*s > 0.05), meaning that participants were more relaxed during this exposure than when they were not exposed to sound.

### Subjective ratings

To investigate the effect of different types of sounds on participants’ perceived state, the *z*‐scored data from the responses to each question were analyzed with a repeated measures ANOVA with sound as the within‐subjects factor (*no sound*, *slow beat*, *brown noise*, *fast beat*).

The ratings for pleasantness and sense of relaxation yielded similar results (Figure 3B,C). Both analyses revealed a significant main effect of sound [pleasantness: *F*_(1, 25)_ = 11.089, *p* < 0.001, $\eta_{p}^{2}$ = 0.307; relaxation: *F*_(1, 25)_ = 4.815, *p* = 0.001, $\eta_{p}^{2}$ = 0.185]. The post hoc comparisons revealed that *slow beat* (pleasantness: *M* = 0.804, SEM = 0.127 and relaxation: *M* = 0.552, SEM = 0.143) was perceived as more pleasant and relaxing than mere silence (pleasantness: *M* = −0.351, SEM = 0.111, *t*_(25)_ = 6.948 *p* < 0.001; relaxation: *M* = −0.202, SEM = 0.146, *t*_(25)_ = 3.058, *p* = 0.016). Moreover, post hoc comparisons of pleasantness showed that *slow beat* was also judged more pleasant than both *fast beat* (*M* = −0.071, SEM = 0.161; *t*_(25)_ = 3.794, *p* = 0.001) and *brown noise* (*M* = −0.380, SEM = 0.168; *t*_(25)_ = 4.580, *p* < 0.001) (Figure 3B). Similarly, for the sense of relaxation, post hoc comparisons also revealed that *slow beat* was judged as more relaxing than *fast beat* music (*M* = −0.448, SEM = 0.164, *t*_(25)_ = 4.388, *p* = 0.001) (Figure 3C). These results indicate that exposure to *slow beat* was the only condition in which participants felt more relaxed and found more pleasant compared to when the sound was not present. Notably, these findings are consistent with the behavioral and physiological results, where this music was found to enhance attention and decrease heart rate.

Post hoc comparisons following the significant main effect of concentration [*F*_(1, 25)_ = 4.347, *p* = 0.006, $\eta_{p}^{2}$ = 0.143] revealed that concentration on the task was easier during the *slow beat* (*M* = 0.200, SEM = 0.148) compared to *no sound* (*M* = −0.499, SEM = 0.156; *t*_(25)_ = 2.830, *p* = 0.02), and concentration on the task was easier during the *fast beat* (*M* = 0.325, SEM = 0.155) compared to *no sound* (*t*_(25)_ = 3.333, *p* = 0.016). There was a significant effect of sound on the level of wakefulness [*F*_(1, 25)_ = 5.723, *p* = 0.001, $\eta_{p}^{2}$ = 0.186], with greater levels in the *fast beat* condition (*M* = 0.598, SEM = 0.140) compared to all the other conditions (*no sound*: *M* = −0.352, SEM = 0.146, *t*_(25)_ = 4.467; *slow beat*: *M* = 0.029, SEM = 0.154, *t*_(25)_ = 2.499; *brown noise*: *M* = −0.275, SEM = 0.181, *t*_(25)_ = 3.098; all *p*s < 0.05). These results highlight that *slow beat* is not only effective at enhancing attention but also at increasing participants’ perception of concentration levels. Interestingly, *fast beat* similarly boosts the sense of concentration, although results about the attentional networks showed no modulation in performance. Thus, this increased sense of focus could be associated with a more energetic and stimulating state, as shown by the increased wakefulness reported in this condition. This interpretation is further supported by the positive Pearson correlation between perceived focus and wakefulness in the *slow beat* condition [*r* = 0.657 *p* < 0.001].

Finally, we also investigated how familiar the stimuli were perceived. No differences were found in familiarity between the stimuli [*F*_(1, 25)_ = 0.719, *p* = 0.544], thus ensuring that the results could not be driven by stimuli familiarity. To summarize, these results indicate that exposure to *slow beat music* increased participants’ attention by strengthening the orienting and executive networks, improving the subjective perception of concentration, and enhancing feelings of pleasure and relaxation, which is further supported by an effective decrease in physiological activation—specifically heart rate.

## DISCUSSION

The present study employed a three‐pronged approach to investigate whether listening to specific sounds or music could improve attentional performance. The results revealed that slow beat music induces relaxation, reduces physiological activation, and, importantly, improves attentional performance while increasing the subjective feeling of concentration and pleasure.

When listening to slow beat music in the background, as compared to the no sound condition, participants were faster in responding to the target if it was preceded by a valid cue. This finding suggests that listening to slow‐tempo music could enhance automatic attention and alertness, thus the ability to respond to exogenous stimuli. As a result, participants might have experienced increased attentional capacity, which allowed them to focus better on the task at hand and respond more effectively to external signals.^4^, ^29^ The observed increase in efficiency for valid cued trials but not invalid cued trials may be linked to the different processes involved in the orienting of attention. Specifically, valid cues are involved in the engagement of attention to the targets, while invalid cues are involved in the disengagement of attention from its current focus (from the target on which it was focused).^30^ The selective decrease of RTs in valid cues, and thus a more efficient attentional engagement, could be related to a general increase in focus and alertness,^31^ which has been shown to be enhanced by background music in low‐demanding, sustained‐attention tasks.^17^ Such enhancement of focus would facilitate the engagement of attention but not its disengagement, as those are two different processes relying on different neural networks.^31^, ^32^

The positive effect of slow beat music was also observed in the executive network, as participants became faster in responding to incongruent trials, in which the target was presented along with distractors. These results indicate that while listening to slow beat music, salient bottom‐up distractors interfered and impacted participants’ performance less than other conditions. In practice, this could be translated into the ability to ignore irrelevant stimuli and focus on the relevant ones.^4^, ^29^ These findings align with recent literature highlighting how attentional networks integrate inputs from multiple sensory modalities and how background noises can affect cognitive performance.^33^, ^34^, ^35^ Furthermore, the present findings contribute to the ongoing debate regarding the potential benefits of auditory stimuli. Certain noises, like brown noise, have no significant effect on attentional efficiency, whereas structured rhythmic input such as slow beat music can positively modulate attention.^6^, ^7^, ^8^ This result contrasts with the sole existing evidence suggesting a positive effect of brown noise on cognitive performance, which, however, was on arithmetic tasks.^8^ In this study, we clarify that brown noise does not exert a similar influence on the attentional networks.

The present study extends this line of research by comparing different types of auditory stimuli and showing that slow‐tempo music also enhances attentional mechanisms—consistent with previous findings^12^—whereas further advancing them by revealing associated physiological modulations that occur independently of emotional content. Indeed, the influence of music has often been explored in relation to psychological states, but its impact on cognitive functions has received less attention.^36^ Our findings expand the understanding of auditory stimulation and suggest that structured, rhythm‐based auditory inputs can significantly modulate attentional and executive efficiency.

Coherently with the enhanced efficacy of attentional processes during slow beat music, positive changes in heart rate (i.e., BPM) were also identified. As compared to the no sound condition, participants’ cardiovascular rhythms decreased when they were listening to slow beat music. This finding aligns with prior research indicating that specific auditory environments can modulate physiological responses such as reducing cortisol levels, heart rate, and blood pressure, and concomitantly alleviating stress and anxiety.^20^, ^22^ A reduction in physiological activation offers substantial benefits to the psycho‐physiological state.^37^ Indeed, stress and high arousal can have effects such as increasing the risk of heart attacks and thus compromising safety and health in everyday life. Over time, they can contribute to acute and chronic issues, such as reduced sleep quality, hypertension, and endocrine disruption.^38^, ^39^ If prolonged, these conditions can increase the long‐term risks of heart disease.^38^, ^40^ Thus, reducing high levels of physiological activation and promoting relaxation through the modulation of cardiovascular rhythms could yield substantial benefits for both health and safety. This is particularly crucial in stressful contexts, such as driving, where the control of top‐down processes and bottom‐up stimuli requires significant cognitive effort.

Although there is data about the positive effect of slow beat music on physiological activation ^22^, the current study provides the first evidence of its impact in combination with increased efficiency of attentional networks as well as within a car‐like environment. Thus, even in a setting reminiscent of a demanding environment in terms of attention and stress, slow beat music is effective in increasing relaxation and suggests that the approach could be successfully applied in these types of challenging environments. The implications of the present study align with previous work,^9^ suggesting that arousal level may mediate the effects of music on cognitive performance. Our findings contribute additional evidence that auditory stimulation—under specific conditions—may influence attention, potentially through changes in arousal. Our findings also offer new insights into how such mechanisms might be leveraged to optimize attentional performance in ecologically valid settings. The fact that Husain and colleagues^9^ found that slow beat music impaired attention, whereas our data show an improvement, may offer new insights into how such mechanisms might be context dependent or exert differential effects on distinct types of attention. In line with the inverted‐U arousal model,^41^, ^42^ we could speculate that the reduction in arousal found in the slow beat condition might have helped counteract stress during the task, potentially contributing to performance improvement. However, future studies should simultaneously measure both attention and arousal to confirm this hypothesis.

In conjunction with a reduced heart rate, participants also explicitly rated slow beat music as more relaxing than all the other conditions. This result indicates that the participants’ feelings of relaxation when listening to slow beat music was not only reflected by the physiological heart rate measure but also subjective psychological experiences. This finding is in agreement with previous data reporting that music around 70 BPM increases relaxation, decreases stress in challenging contexts, and improves sleep quality.^36^, ^43^ This increase in participants’ sense of relaxation was accompanied by an increased sense of pleasure, which aligns with previous studies that have also revealed positive emotions induced by listening to music.^21^, ^22^, ^23^ This finding expands upon previous research suggesting that music with positive valence can modulate selective attention via specific brain networks^10^ by adding information regarding the link with physiological activation. Furthermore, these findings contribute to the discussion on the role of pleasure in response to auditory stimuli and how tempo preference may play a role in cognitive and emotional responses to music.^16^, ^17^

Although slow rhythms are known to align with the natural rhythms of the human body and induce a calming effect,^22^ fast beat music is known to increase the feeling of excitement and performance during physical exercise.^25^, ^44^ It is therefore possible that in the car‐like setting where participants were seated, the calming effects of slower music might have been particularly appealing. Additionally, the dynamic attending theory suggests that external rhythmic stimuli can entrain internal attentional oscillations and enhance temporal prediction and attentional focus.^45^, ^46^ In the context of our study, slower rhythmic patterns may have more readily entrained internal attentional rhythms, potentially enhancing not only cognitive performance but also the perceived coherence and pleasantness of the auditory experience. Interestingly, this sense of pleasure was not linked to the familiarity of the stimuli. Previous research has shown that familiarity with a melody can modulate cognitive processing and attentional engagement.^47^ However, our findings indicate that the benefits of slow‐tempo music on attention cannot be driven by familiarity with the type of sounds, reinforcing the idea that tempo and rhythmic structure are key factors in modulating not only the subjective response but also attentional performance and physiological activation. To confirm this hypothesis, future studies should simultaneously measure both attention and arousal. Moreover, beyond familiarity, participants’ musical background and listening habits might play a role in modulating both their performance and stress, and future studies should consider taking these factors into account as well.

Interestingly, the music conditions were the only ones that increased the sense of focus perceived by participants. Although an increase in perceived concentration has been associated with increased performance and self‐efficacy,^48^, ^49^ there is no evidence in the literature of related changes in attention level. In the present study, only slow beat music effectively increased attention efficiency. During the fast beat music exposure, the sense of increased focus was associated with enhanced level of wakefulness but did not impact attentional performance or physiological activation. Although previous data showed that this type of music has an energizing and stimulating effect,^25^, ^36^, ^44^ this study is the first to examine fast beat music with levels of wakefulness. This subjective perception is particularly relevant in contexts such as cycling, driving, or other activities requiring a high level of alertness, where sleepiness poses a significant threat to an individual's ability to remain focused on tasks and the surrounding.^50^ Thus, fast tempos could potentially reduce fatigue during prolonged periods of activity. It is also relevant to note that its stimulating properties may enhance alertness without inducing stress or increased physiological activation and therefore will likely not compromise an individual's health. However, further investigations are needed to test our speculation.

It is important to consider that, given the high number of conditions to compare, we opted for the shorter version of the ANT to prevent the study from becoming excessively long and minimize the influence of fatigue on participants. Future studies should consider using the complete version and investigate other neural attentional networks, such as the alerting network. Nevertheless, the absence of perceptual loudness control across conditions could have influenced the study results.

In summary, our study provides new evidence about the influence of auditory stimuli on visual attention^51^ and highlights the positive impact of slow‐tempo music on automatic attention and executive attentional network. It also shows a reduction in physiological activation, as measured by heart rate, and an increased feeling of relaxation, pleasure, and focus. This research is the first to compare the effects of different types of music and noise on cognitive, physiological, and subjective responses, showing that slow‐beat music can be a simple yet effective approach to reduce cognitive overload and enhance performance in various contexts.

## AUTHOR CONTRIBUTIONS

Lucia De Francesco, Alessandro Mazza, Raffaella Ricci, and Olga Dal Monte designed the study. Lucia De Francesco performed the experiments. Lucia De Francesco and Alessandro Mazza pre‐processed the physiological data. Lucia De Francesco analyzed the data. Lucia De Francesco, Selene Schintu, and Olga Dal Monte wrote the article. All authors reviewed the manuscript.

## CONFLICT OF INTEREST STATEMENT

The authors declare no conflicts of interest.

## Peer Review

The peer review history for this article is available at https://publons.com/publon/10.1111/nyas.70049

## Data Availability

The data that support the findings of this study are openly available in Sounds_Attention at https://github.com/SocialInteractionLabUnito/Sounds_Attention.

## References

[1] Desimone, R. , & Duncan, J. (1995). Neural mechanisms of selective visual attention. Annual Review of Neuroscience, 18, 193–222. 10.1146/annurev.ne.18.030195.001205 7605061

[2] Shomstein, S. , & Yantis, S. (2004). Control of attention shifts between vision and audition in human cortex. The Journal of Neuroscience, 24(47), 10702–10706. 10.1523/JNEUROSCI.2939-04.2004 15564587 PMC6730120

[3] Tarabay, R. , & Abou‐Zeid, M. (2018). Assessing the effects of auditory‐vocal distraction on driving performance and physiological measures using a driving simulator. Transportation Research Part F: Traffic Psychology and Behaviour, 58, 351–364. 10.1016/j.trf.2018.06.026

[4] Weaver, B. , Bédard, M. , McAuliffe, J. , & Parkkari, M. (2009). Using the attention network test to predict driving test scores. Accident; Analysis and Prevention, 41(1), 76–83. 10.1016/j.aap.2008.09.006 19114140

[5] Welz, W. , Voelter‐Mahlknecht, S. , Große‐Siestrup, C. , & Preuß, G. (2020). The influence of different auditory stimuli on attentiveness and responsiveness in road traffic in simulated traffic situations. International Journal of Environmental Research and Public Health, 17(24), 9226. 10.3390/ijerph17249226 33321821 PMC7764073

[6] Awada, M. , Becerik‐Gerber, B. , Lucas, G. , & Roll, S. (2022). Cognitive performance, creativity and stress levels of neurotypical young adults under different white noise levels. Scientific Reports, 12(1), 14566. 10.1038/s41598-022-18862-w 36028546 PMC9418159

[7] Guo, K. , Wu, Y. , & Zhang, H. (2022). The effects of color noises on attention. In Proceedings of the 2022 International Conference on Science Education and Art Appreciation (pp. 576–583). Atlantis Press. 10.2991/978-2-494069-05-3_71

[8] Proverbio, A. M. , Benedetto, F. D. , Ferrari, M. V. , & Ferrarini, G. (2018). When listening to rain sounds boosts arithmetic ability. PLoS ONE, 13(2), e0192296. 10.1371/journal.pone.0192296 29466472 PMC5821317

[9] Husain, G. , Thompson, W. F. , & Schellenberg, E. G. (2002). Effects of musical tempo and mode on arousal, mood, and spatial abilities. Music Perception: An Interdisciplinary Journal, 20(2), 151–171. 10.1525/mp.2002.20.2.151

[10] Fernandez, N. B. , Trost, W. J. , & Vuilleumier, P. (2019). Brain networks mediating the influence of background music on selective attention. Social Cognitive and Affective Neuroscience, 14(12), 1441–1452. 10.1093/scan/nsaa004 31993668 PMC7137722

[11] Finucane, A. M. , Whiteman, M. C. , & Power, M. J. (2010). The effect of happiness and sadness on alerting, orienting, and executive attention. Journal of Attention Disorders, 13(6), 629–639. 10.1177/1087054709334514 19448148

[12] Dovorany, N. , Brannick, S. , Johnson, N. , Ratiu, I. , & LaCroix, A. N. (2023). Happy and sad music acutely modulate different types of attention in older adults. Frontiers in Psychology, 14, 1029773. 10.3389/fpsyg.2023.1029773 36777231 PMC9909555

[13] Marti‐Marca, A. , Nguyen, T. , & Grahn, J. A. (2020). Keep calm and pump up the Jams: How musical mood and arousal affect visual attention. Music & Science, 3, 2059204320922737. 10.1177/2059204320922737

[14] Ünal, A. B. , Steg, L. , & Epstude, K. (2012). The influence of music on mental effort and driving performance. Accident; Analysis and Prevention, 48, 271–278. 10.1016/j.aap.2012.01.022 22664690

[15] Unal, A. B. , de Waard, D. , Epstude, K. , & Steg, L. (2013). Driving with music: Effects on arousal and performance. Transportation Research. Part F: Traffic Psychology and Behaviour, 21, 52–65. 10.1016/j.trf.2013.09.004

[16] Dobbs, S. , Furnham, A. , & McClelland, A. (2011). The effect of background music and noise on the cognitive test performance of introverts and extraverts. Applied Cognitive Psychology, 25(2), 307–313. 10.1002/acp.1692

[17] Kiss, L. , & Linnell, K. J. (2021). The effect of preferred background music on task‐focus in sustained attention. Psychological Research, 85(6), 2313–2325. 10.1007/s00426-020-01400-6 32748062 PMC8357712

[18] De Francesco, L. , Mazza, A. , Sorrenti, M. , Murino, V. , Battegazzorre, E. , Strada, F. , Bottino, A. G. , & Dal Monte, O. (2024). Cooperation and competition have same benefits but different costs. Iscience, 27(7), 110292. 10.1016/j.isci.2024.110292 39045102 PMC11263633

[19] Fujimura, T. , Katahira, K. , & Okanoya, K. (2013). Contextual modulation of physiological and psychological responses triggered by emotional stimuli. Frontiers in Psychology, 4, 212. 10.3389/fpsyg.2013.00212 23675359 PMC3650463

[20] Fukui, H. , & Yamashita, M. (2003). The effects of music and visual stress on testosterone and cortisol in men and women. Neuro Endocrinology Letters, 24(3–4), 173–180. 14523353

[21] Pelletier, C. L. (2004). The effect of music on decreasing arousal due to stress: A meta‐analysis. Journal of Music Therapy, 41(3), 192–214. 10.1093/jmt/41.3.192 15327345

[22] Knight, W. E. , & Rickard, N. S. (2001). Relaxing music prevents stress‐induced increases in subjective anxiety, systolic blood pressure, and heart rate in healthy males and females. Journal of Music Therapy, 38(4), 254–272. 10.1093/jmt/38.4.254 11796077

[23] Tansik, D. A. , & Routhieaux, R. (1999). Customer stress‐relaxation: The impact of music in a hospital waiting room. International Journal of Service Industry Management, 10(1), 68–81. 10.1108/09564239910255389

[24] Labbé, E. , Schmidt, N. , Babin, J. , & Pharr, M. (2007). Coping with stress: The effectiveness of different types of music. Applied Psychophysiology and Biofeedback, 32(3), 163–168. 10.1007/s10484-007-9043-9 17965934

[25] Swaminathan, S. , & Schellenberg, E. G. (2015). Current Emotion research in music psychology. Emotion Review, 7(2), 189–197. 10.1177/1754073914558282

[26] Cheah, Y. , Wong, H. K. , Spitzer, M. , & Coutinho, E. (2022). Background music and cognitive task performance: A systematic review of task, music, and population impact. Music & Science, 5, 20592043221134392. 10.1177/20592043221134392

[27] Weaver, B. , Bédard, M. , & McAuliffe, J. (2013). Evaluation of a 10‐minute version of the attention network test. The Clinical Neuropsychologist, 27(8), 1281–1299. 10.1080/13854046.2013.851741 24205860

[28] Kim, H.‐G. , Cheon, E.‐J. , Bai, D.‐S. , Lee, Y. H. , & Koo, B.‐H. (2018). Stress and heart rate variability: A meta‐analysis and review of the literature. Psychiatry Investigation, 15(3), 235–245. 10.30773/pi.2017.08.17 29486547 PMC5900369

[29] Zamani Sani, S. H. , Fathirezaie, Z. , Sadeghi‐Bazargani, H. , Badicu, G. , Ebrahimi, S. , Grosz, R. W. , Sadeghi Bahmani, D. , & Brand, S. (2020). Driving accidents, driving violations, symptoms of attention‐deficit‐hyperactivity (ADHD) and attentional network tasks. International Journal of Environmental Research and Public Health, 17(14), 5238. 10.3390/ijerph17145238 32698490 PMC7400088

[30] Posner, M. I. (1980). Orienting of attention. The Quarterly Journal of Experimental Psychology, 32(1), 3–25. 10.1080/00335558008248231 7367577

[31] Posner, M. I. , & Petersen, S. E. (1990). The attention system of the human brain. Annual Review of Neuroscience, 13, 25–42. 10.1146/annurev.ne.13.030190.000325 2183676

[32] Petersen, S. E. , & Posner, M. I. (2012). The attention system of the human brain: 20 years after. Annual Review of Neuroscience, 35, 73–89. 10.1146/annurev-neuro-062111-150525 PMC3413263 22524787

[33] Fan, Y. , Liang, J. , Cao, X. , Pang, L. , & Zhang, J. (2022). Effects of noise exposure and mental workload on physiological responses during task execution. International Journal of Environmental Research and Public Health, 19(19), 12434. 10.3390/ijerph191912434 36231736 PMC9566815

[34] Hammer, M. S. , Swinburn, T. K. , & Neitzel, R. L. (2014). Environmental noise pollution in the United States: Developing an effective public health response. Environmental Health Perspectives, 122(2), 115–119. 10.1289/ehp.1307272 24311120 PMC3915267

[35] Jafari, M. J. , Khosrowabadi, R. , Khodakarim, S. , & Mohammadian, F. (2019). The effect of noise exposure on cognitive performance and brain activity patterns. Open Access Macedonian Journal of Medical Sciences, 7(17), 2924–2931. 10.3889/oamjms.2019.742 31844459 PMC6901841

[36] Iyendo, T. O. (2016). Exploring the effect of sound and music on health in hospital settings: A narrative review. International Journal of Nursing Studies, 63, 82–100. 10.1016/j.ijnurstu.2016.08.008 27611092

[37] Alsuraykh, N. H. , Wilson, M. L. , Tennent, P. , & Sharples, S. (2019). How stress and mental workload are connected. In Proceedings of the 13th EAI International Conference on Pervasive Computing Technologies for Healthcare (pp. 371–376). ACM Digital Library. 10.1145/3329189.3329235

[38] Basner, M. , Babisch, W. , Davis, A. , Brink, M. , Clark, C. , Janssen, S. , & Stansfeld, S. (2014). Auditory and non‐auditory effects of noise on health. The Lancet, 383(9925), 1325–1332. 10.1016/S0140-6736(13)61613-X PMC3988259 24183105

[39] Meiring, G. A. M. , & Myburgh, H. C. (2015). A review of intelligent driving style analysis systems and related artificial intelligence algorithms. Sensors, 15(12), 30653–30682. 10.3390/s151229822 26690164 PMC4721742

[40] Chung, W.‐Y. , Chong, T.‐W. , & Lee, B.‐G. (2019). Methods to detect and reduce driver stress: A review. International Journal of Automotive Technology, 20(5), 1051–1063. 10.1007/s12239-019-0099-3

[41] Beerendonk, L. , Mejías, J. F. , Nuiten, S. A. , de Gee, J. W. , Fahrenfort, J. J. , & van Gaal, S. (2024). A disinhibitory circuit mechanism explains a general principle of peak performance during mid‐level arousal. Proceedings of the National Academy of Sciences, 121(5), e2312898121. 10.1073/pnas.2312898121 PMC10835062 38277436

[42] Yerkes, R. M. , & Dodson, J. D. (1908). Classics in the history of psychology. York University. https://psychclassics.yorku.ca/Yerkes/Law/

[43] Lai, H.‐L. , & Good, M. (2006). Music improves sleep quality in older adults. Journal of Advanced Nursing, 53(1), 134–144. 10.1111/j.1365-2648.2006.03693.x 16422710

[44] Karageorghis, C. I. , & Terry, P. (1997). The psychophysical effects of music in sport and exercise: A review. Journal of Sport Behavior, 20, 54–68.

[45] Jones, M. R. (1976). Time, our lost dimension: Toward a new theory of perception, attention, and memory. Psychological Review, 83(5), 323–355. 10.1037/0033-295X.83.5.323 794904

[46] Large, E. W. , & Jones, M. R. (1999). The dynamics of attending: How people track time‐varying events. Psychological Review, 106(1), 119–159. 10.1037/0033-295X.106.1.119

[47] Pereira, C. S. , Teixeira, J. , Figueiredo, P. , Xavier, J. , Castro, S. L. , & Brattico, E. (2011). Music and emotions in the brain: Familiarity matters. PLoS ONE, 6(11), e27241. 10.1371/journal.pone.0027241 22110619 PMC3217963

[48] de Bree, E. , & Zee, M. (2021). The unique role of verbal memory, vocabulary, concentration and self‐efficacy in children's listening comprehension in upper elementary grades. First Language, 41(2), 129–153. 10.1177/0142723720941680

[49] Wolfgramm, C. , Suter, N. , & Göksel, E. (2016). Examining the role of concentration, vocabulary and self‐concept in listening and reading comprehension. International Journal of Listening, 30(1–2), 25–46. 10.1080/10904018.2015.1065746

[50] Moradi, A. , Nazari, S. S. H. , & Rahmani, K. (2019). Sleepiness and the risk of road traffic accidents: A systematic review and meta‐analysis of previous studies. Transportation Research Part F: Traffic Psychology and Behaviour, 65, 620–629. 10.1016/j.trf.2018.09.013

[51] Chillemi, G. , Calamuneri, A. , Quartarone, A. , Terranova, C. , Salatino, A. , Cacciola, A. , Milardi, D. , & Ricci, R. (2019). Endogenous orientation of visual attention in auditory space. Journal of Advanced Research, 18, 95–100. 10.1016/j.jare.2019.01.010 30828479 PMC6383076

